# Effectiveness of Folic Acid Supplementation Recommendations among Polish Female Students from the Podkarpackie Region

**DOI:** 10.3390/nu13031001

**Published:** 2021-03-19

**Authors:** Maria Zadarko-Domaradzka, Ewa Kruszyńska, Emilian Zadarko

**Affiliations:** 1 Institute of Physical Culture Studies, Medical College of Rzeszów University, University of Rzeszów, 35-959 Rzeszów, Poland; mzadarko@ur.edu.pl; 2 Faculty of Physical Culture and Health, University of Szczecin, 70-453 Szczecin, Poland; ewa.kruszynska@usz.edu.pl

**Keywords:** public health policy, supplementation, folic acid, prevention

## Abstract

Adequate folic acid supplementation during the preconception period is an important element in the primary prevention of neural tube defects (NTDs). This study aims to study the effectiveness of folic acid supplementation recommendations among women of childbearing age, and to assess and characterise their awareness about this public health measure. The cross-sectional study included women (*N* = 1285) aged 22.27 ± 4.6 years old on average. Some of the results were obtained on a subgroup of women (*N* = 1127) aged 21.0 ± 2.1. This study was performed using a questionnaire. The analysis was performed with the use of a logistic regression model, chi-square test for independence and odds ratio (OR). According to the results, only 13.9% of women supplement folic acid, and 65.3% of them do so daily. A total of 91.1% of the respondents were not aware of its recommended dose and 43% did not know the role it plays in the human body. Among women who do not currently supplement folic acid (*N* = 1052), 52.4% declared doing so while planning their pregnancy. Women’s awareness about the role of folic acid in NTD prevention (OR = 4.58) and the information they got from physicians (OR = 1.68) are key factors that increased the odds of the women taking folic acid before pregnancy. There is therefore a need for more information and education campaigns to raise awareness about folic acid.

## 1. Introduction

Folic acid intake is an important public health goal that can reduce the risk of neural tube defects (NTDs) [1]. This B-group vitamin occurs naturally in green leafy vegetables, citrus fruits and legumes, as well as in animal source foods, such as eggs and liver [2,3,4]. The supply of naturally occurring folates through food is possible and, sometimes, sufficient, however, their absorption from food can be limited, as these compounds are very sensitive to many factors, including high temperature [3,5].

A synthetic form of naturally occurring folate is used in supplements and in fortified foods [2]. Over several years, many studies have corroborated that preconception supplementation with folic acid reduces the risk of developing neural tube defects (e.g., anencephaly, spina bifida) [1,6,7,8,9]. NTDs are congenital malformations that originate during early embryonic development when the neural tube fails to close completely, which results in perinatal mortality or disabilities; thus, they constitute an important problem in public health on a global level [5,10,11]. The key aspect of active NTD prevention is the period in which folic acid supplementation is introduced. For folic acid to fulfil its preventive role and be used in the right period, it is necessary that women plan their pregnancy and learn about folic acid before becoming pregnant [12,13]. The World Health Organization (WHO) recommends that “[a]ll women, from the moment they begin trying to conceive until 12 weeks of gestation, should take a folic acid supplement (400 μg folic acid daily)” [14]. In 2020, the Polish Society of Gynaecologists and Obstetricians released new recommendations, including (p. 651) “According to the up-to-date knowledge, it is recommended to: Use 0.4 mg/d of folic acid in all women of reproductive age, as a supplement to a natural, folate-rich diet” [15]. Prior to this, in 2017, it was recommended that women of childbearing age supplement this vitamin for at least 12 weeks before conception, as well as include folate-rich products and fortified foods in their daily diet [16].

In Poland, as in all European Union (EU) countries, there is no mandatory food fortification [17,18]; however, the Polish market currently offers food products fortified with folic acid; mainly breakfast cereals, juices, and nectars [19].

Studies by Khoshnood et al. (2015) show that, in Europe, despite long-term recommendations for dietary supplementation with folic acid during the preconception period, the incidence of neural tube defects has not decreased. Between 1991 and 2011, the overall, total prevalence of NTDs was of a similar magnitude across Europe, at 9.1 per 10,000 births. The incidence of NTDs in Poland in the Wielkopolska region was 9.25 per 10,000 births [20]. Such findings are also corroborated by the JRC-EUROCAT report. The frequency of NTDs in Europe has not decreased over the last 10 years. Prevalence rates were 10.10 per 10,000 births in the years 2006–2007 and 10.27 per 10,000 births in the years 2014–2015 [21]. According to 2018 data, the rate was 10.75 per 10,000 births [22]. Cross-sectional studies (*N* = 466,860) of women from England and the Isle of Man between 1999 and 2012 show that a relatively small group of women (6%) aged under 20, consume folic acid supplements prior to pregnancy [17]. Studies by Kinunnen et al. (2017) indicate that 30.1% of women in Europe use folic acid supplements before pregnancy [23].

According to studies conducted in Poland among pregnant women in 2010–2012 (*N* = 8625) and in 2017 (*N* = 3441), only one in three women surveyed was aware of the importance of folic acid supplementation before conception and the recommended dose, and most women (about 62%) start taking folic acid after pregnancy is confirmed [24]. Another study shows that 42% of the surveyed women took folic acid before pregnancy. When pregnant, folic acid was supplemented by 83% of women, of which 71% started as late as the fifth–sixth week of pregnancy and beyond [25]. Meanwhile, a survey among female non-medical students (*N* = 325) showed that in 2008, 35.3% of them were aware of the importance of periconceptional folic acid supplementation, and in 2013, the figure reached 41.1% [26].

In 1998, the Polish government implemented a nationwide programme titled “Primary Prophylaxis of Neural Tube Defects” (PPNTDs). The programme recommended that all women of childbearing age in Poland who are likely to become pregnant consume 0.4 mg of folic acid daily in order to reduce the risk of neural tube defects in their offspring. Moreover, the aim of the programme was to promote society-wide awareness of folic acid and its relation to NTDs. It was expected to influence attitudes and shape proper behaviours regarding folic acid supplementation among women. The idea was that the programme would be introduced into health education projects and also implemented in health care facilities providing services to mothers-to-be and pregnant women. As part of this, activities and educational materials have been designed for young people aged 16–24 entitled “I can provide for the health of my future child right now” and educational videos called “A chance for life” and “It is just enough to want to”. In addition, the programme was to be distributed by campaigns on all types of mass media. The recommendations of the Polish PPNTDs programme were developed by a team of experts appointed by the Minister of Health and Social Welfare [27,28]. Although the programme was established more than 20 years ago, the recommended intake level of folic acid for women of childbearing age is in line with the current guidelines of the WHO and the Polish Society of Gynaecologists and Obstetricians [14,15].

Monitoring preventive behaviours, as well as diagnosing the state of knowledge, seems to be an important element of assessing the effectiveness of current measures within health policies, including reproductive health [24,29,30]. It is important for women to be aware of folic acid supplementation in order to start consuming folic acid [31].

This study aims to study the effectiveness of folic acid supplementation recommendations among women of childbearing age and also to assess and characterise their awareness about this public health measure.

## 2. Materials and Methods

The data on folic acid supplementation and knowledge of folic acid were obtained using an online questionnaire completed by women studying in public universities in major cities (Rzeszów, Krosno, Sanok) of the Podkarpackie Voivodeship. Podkarpackie is one of the 16 voivodeships, being the most southern one in Poland.

A questionnaire was created for the purpose of this study with questions (supplement file 1) based on the educational materials of the programme for young people aged 16–24, entitled ‘I can provide for the health of my future child right now’, which was developed within the PPNTDs project [28]. In order to optimise the questionnaire with respect to the content, layout and the understanding of the questions a pilot test was conducted in a group (*N* = 25) representing the target population, yet, not included in the main study. There were no structural errors found in the survey.

The data presented below were gathered from 1285 women in the age range of 19–40, whose average age was 22.27 ± 4.6 years old. The vast majority were women up to 30 years old. There were 97 participants over the age of 30. The data were collected between 2016 and 2017. In sampling, the statistical error for the sampling fraction was assumed to be no greater than 3%. With the assumption of the size of the target population at the level of 28.000, this gives the minimal size of the sample of 1028 people. As more questionnaires were collected during the course of the study, they were included in the analysis. According to the Statistical Office in Rzeszów, in the academic year 2016/2017 at the universities of Podkarpackie women constituted 55.1% of all the students (51.051), which is 28.127 people [32].

During obligatory physical education classes for different courses, qualified interviewers encouraged women to become part of the study and answered all of their questions related to filling in the electronic version of the questionnaire on mobile devices. The link to the questionnaire could be found on the website of the Department of Health Sciences of the Faculty of Physical Education at the University of Rzeszów. Each participant received a questionnaire that contained information on the purpose of the study and questions regarding their knowledge of folic acid and its supplementation, frequency of consumption of green leafy vegetables, as well as socio-demographic questions involving age, body weight and height, place of residence (city, village), and the number of children. The criteria for the participation of the women (students) in this study included voluntary consent to participate in the study (by accessing the link and sending the anonymously completed online questionnaire) and full completion of all of the items in the questionnaire. All collected data were sent anonymously and voluntarily by the respondents. The study was performed in accordance with the ethical standards defined in the Declaration of Helsinki. Permission of the Bioethics Committee 20 December, 2015—University of Rzeszów.

The analysis of the collected data was prepared in two stages. Firstly, summaries were made using descriptive statistics (numerical and percentage summaries). They included the knowledge and behaviour declared by all women participating in the study. Then, women without children, not currently pregnant or up to 30 years old (*N* = 1127) were selected from the study group (*N* = 1285) and this group was subjected to a detailed analysis. The methods included a logistic regression model, chi-square test for independence and *OR*–odds ratio (with a 95% confidence interval). Multiple logistic regression was used to model the probability of the intention to take folic acid before pregnancy as a function of factors associated with knowledge regarding folic acid. Place of residence and Body Mass Index (BMI) were introduced as control factors into the model.

Statistical significance was taken as *p* < 0.05. For some of the tables, including low-number cells, the results of the chi-squared test were verified by means of the Fisher exact test.

## 3. Results

### 3.1. Women’s Knowledge and Behaviour in the Surveyed Population N = 1285

The percentage distribution of the participants, in terms of their place of residence, was as follows (54.6% village, 45.4% city). The participants of the study were aged between 19 and 40 years. The mean age equalled 22.27 ± 4.6 years old. Female students over the age of 30 made up 7.6% of all respondents. An overwhelming 89.9% of the surveyed women did not have children yet (Table 1).

A total of 90% of the women declared that they have heard about folic acid, but only 14.0% of them knew that it is a water-soluble vitamin. Only 8.9% of the respondents were aware of the folic acid dose that is recommended for women of reproductive age. Moreover, 40.6% of the surveyed women were familiar with the role of folic acid in the prevention of neural tube defects, but 43% of them knew nothing about the role it plays in the human body. Among all of the respondents, 35% could not indicate good sources of folate in food. Only 41.6% knew that it occurs in green leafy vegetables, 32.5% indicated liver, and 18.8% citrus fruit.

A total of 86.1% of women did not use any folic acid supplements. Among women who supplemented folic acid, 65.3% did so daily, as recommended. Most surveyed women declared that they did not supplement folic acid because there was no need to do so (55.5%) or because they lacked any knowledge of folic acid (20.8%). Some indicated that it was not important for them (6.8%) and that they did not know the reason that they did not use the supplement (25.7%). Only 52.4% of those surveyed were going to take folic acid while planning a pregnancy, 42.3% were not sure, and 5.2% did not intend to do so. More than half of the respondents (53%) indicated the internet as their source of knowledge about folic acid, 27.2% of the surveyed women pointed out their physician, and 22.6%, their school/university (Supplement Table S1).

### 3.2. Selected Group of Women: Without Children and up to 30 Years Old N = 1127

Women without any children, not pregnant at the time, and up to 30 years old were selected (Supplement Table S2) from the whole study population to unify the group (according to Eurostat, in 2018, Polish women had their first baby at the age of 27.4) [34]. Table 2 shows the general characteristics of the selected population.

Table 3 shows the respondents’ statement on folic acid supplementation, followed by the reason for its intake or lack thereof.

One of the subjects of the analysis was the correlation between the knowledge about folic acid and its intake. It involved a comparison of the frequency of the respective answers to the questions, regarding basic knowledge in the group of women who supplement folic acid and those who do not. Table 4 shows the number and percentage of women from both groups and their respective answers. The significance of the differences between the groups was assessed with the use of the chi-square test for independence.

The women who use folic acid supplements have greater knowledge about their role in the prevention of neural tube defects than those who do not use such supplementation (*p* ≤ 0.001). Many respondents search for information about folic acid on the internet, however, this does not differentiate behaviours related to its intake in a significant way.

The women were also asked about the frequency of consumption of green leafy vegetables. Of the examined women, 3.7% admitted to the daily consumption of green leafy vegetables. The analysis did not show a statistically significant (*p* = 0.334) difference in the frequency of consumption of green leafy vegetables among women using and not using supplements (Supplement Tables S3 and S4).

### 3.3. Modelling the Intention to Consume Folic Acid before Pregnancy (N = 1052)

Considering selected factors concerning key aspects of knowledge about folic acid, knowledge about the prevention of neural tube defects, the ability to indicate at least one food product as a source of folate, and the source of the knowledge, a multiple logistic regression model was constructed in which the intention to take folic acid before pregnancy was a dependent variable. Place of residence and BMI were also introduced as control factors into the model, but did not prove statistically significant and were not included in the final version of the model. The analysis involved the group of 1052 women who declared that they did not use folic acid supplementation (see Table 5). All variables included in the model were statistically significant.

It may be observed that all factors concerned increase the odds of women taking folic acid before becoming pregnant. The knowledge about the prevention of NTDs seems to be of utmost importance. It increases the odds of women deciding to take folic acid approximately 4.58 times (OR = 4.58 95%, CI = 3.39–6.18). The other two factors are of less importance, but they are still relevant: knowledge about products rich in folates (OR = 1.84 95% CI = 1.39–2.43) and physician as a source of knowledge about folic acid (OR = 1.68 95% CI = 1.22–2.30).

### 3.4. Depth of Knowledge about Folic Acid vs. Knowledge about Prevention of Neural Tube Defects

It was also analysed if the so-called depth of knowledge (awareness of the recommended daily dose of folic acid, ability to name some products rich in folates, or physician as a source of knowledge) is greater among women who know that folic acid helps prevent neural tube defects. Table 6 shows the number and percentage of women with so-called “deep” knowledge, depending on their knowledge concerning the prevention of neural tube defects.

It has been observed that knowledge of the role of folic acid in the prevention of neural tube defects is related to the selected elements of deep knowledge (recommended intake level and good sources of folate in the diet).

## 4. Discussion

One of the most important vitamins in a woman’s diet before and at the early stage of pregnancy is a B-group vitamin: folic acid. A total of 90% of our study participants have heard of folic acid, which is comparable to the corresponding rate observed in a Japanese study (91.2%) [35]. A survey carried out among 1285 women of reproductive age has shown that a big part of the studied population is not aware of the need for folic acid supplementation, especially in the preconception period, and is not familiar with its food sources. Among the women who do not take folic acid (86.1%), only half of them (52.4%) intend to take it before pregnancy, 42.3% of the women surveyed are not sure, and 5.2% say they do not plan on taking a folic acid supplement. Folate deficiency is associated with various adverse health effects, including complications of pregnancy, such as neural tube defects, intrauterine growth restriction and recurrent pregnancy loss [36]. This may be caused by an insufficient dietary folate intake, impaired folate absorption or metabolism, as well as increased demand for folates, for instance during pregnancy [37]. It is estimated that young non-pregnant women in Poland (aged 18–35) take about 127–315 mcg of folic acid per person per day [38]. According to data published in 2018, pregnant women in Poland consume an average of 254 mcg of folic acid per day in their diet [39]. In the countries where food fortification is not mandatory, daily dietary supplementation with folic acid is required at a dose of 400 mcg per person per day, to maintain healthy levels of this vitamin in the body. Effective intervention programmes are therefore needed to improve folic acid intake in the preconception period [40]. Just as in our study, other authors see the effectiveness of the educational programme for the prevention of neural tube defects (NTDs) in Poland as unsatisfactory [26]. According to the EUROCAT report for Poland, the proportion of pregnancies which are planned in Poland is low and the proportion of women taking folic acid before pregnancy was found to be 5.5% in 2003, 7.4% in 2004, and 10.6% in 2005 [41]. Studies published between 2000 and 2010 show that only about 18–25% of young non-pregnant women in Poland declared folic acid supplementation [42]. Subsequent Polish studies on folic acid supplementation by women in Poland, published in 2011–2018, do not indicate any improvement in behaviour in this regard. Among a Lower Silesia study group of women who became mothers (*N* = 894), 17.8% confirmed that they took folic acid before pregnancy [43]. According to a survey conducted in Warsaw in a group of 120 women with a mean age of 24.91, not pregnant at the time of the survey, and with no history of pregnancy, it was shown that one in four were taking a dietary supplement containing folic acid, and 22% of the respondents were familiar with the Polish NTD Primary Prevention programme [44]. A 2016 survey of female medical students (*N* = 276) observed low folic acid supplementation. Only 14.9% were taking it regularly [45]. In 2018, 5.6% of female medical students (*N* = 125, mean age 21.3 years) supplemented folic acid [46]. The results of our study among female students from the Podkarpackie region (*N* = 1285) are consistent with the findings of other studies conducted in different parts of Poland. A total of 13.9% of women supplement folic acid, of which 6.9% take the supplement only when pregnant. In the group of women with no children and aged up to 30 years old (*N* = 1127), 6.7% take folic acid, of which 44% do so on a daily basis. A programme designed by the Polish Ministry of Health titled “A programme on complex reproductive healthcare in Poland between 2016 and 2020” points out (p. 7) the fact that “[m]any sexually active women of childbearing age do not consume proper doses of folic acid”; however, it does not offer any solution to remedy this situation [47].

The multivariable analysis presented in our results showed that the women’s awareness of folic acid’s role in reducing the risk of NTDs (OR = 4.58), the source of the knowledge being the physician (OR = 1.68) and the knowledge of products rich in folates (OR = 1.84) are the significant factors associated with women taking folic acid before pregnancy. The majority of the surveyed women seek knowledge on the internet; however. it is the information provided by the physician that may encourage appropriate health behaviours among women. Similar results were obtained in a 2018 study among Japanese women. There, too, the most common source of information about folic acid was the internet, and among women taking folic acid, were those who received information from medical personnel. This study also revealed that awareness of the role of folic acid in the prevention of NTDs was a variable associated with periconceptional folic acid supplementation (OR = 1.75) [35]. Our findings show that there is a huge knowledge deficit among young Polish women in terms of folic acid and its role in the prevention of neural tube defects, as well as a lack of appropriate behaviour associated with the recommendations for preconception supplementation. Results showed that 40.6% of the surveyed women are familiar with the role of folic acid in the prevention of neural tube defects.

The results of a study carried out among young women in Slovakia also showed the limited knowledge of the influence of folic acid on embryonic development in women of childbearing age [40]. All women of reproductive age (12–45 years old) and fertile (it is possible for them to get pregnant) should be informed about the beneficial effects of folic acid obtained through multivitamin supplementation [48]. A study by Friberg (2015) showed that despite the domestic recommendations implemented in Denmark regarding preconception supplementation with folic acid, women do not follow the guidelines. Women of low socio-economic status are particularly likely to lack information about folic acid supplementation with regard to pregnancy [49]. Moreover, studies performed in Italy showed that only 23.5% of the surveyed women consumed folic acid supplements preconception. A significantly higher percentage of women (54.9%) started the supplementation after the confirmation of pregnancy, that is, during the first trimester [18]. However, studies show that a significant number of women do not follow the recommendations for prophylactic folic acid, most often starting the supplementation too late, only after the pregnancy is already diagnosed [24].

Mills (2017) observes that if pregnancies are unplanned and women who may potentially become pregnant do not use supplementation, it is difficult, at the population level, for folic acid to reduce the risk of having a child with a neural tube defect due to the fact that neural tube closure is completed 28 days from conception, before most women learn about their pregnancy [50]. According to the findings, 40–44% of all pregnancies globally are unplanned [51,52]. Since measures that involve recommendations for folic acid supplementation in the preconception period are insufficient and contribute to health inequalities, it is necessary to introduce food fortification because the incidence of neural tube defects has not decreased in Europe [17,20,53]. The results of research from many countries show that programmes recommending folic acid supplementation have failed, and such factors, as the young age of the mother, unplanned pregnancy, lower levels of education, and the lack of a partner, significantly correlate with the lack of appropriate supplementation practices [11,17,35,54].

In 1992, the United States Public Health Service established recommendations on folic acid supplementation. From 1998, it has also mandated folic acid fortification of flour [10]. Over the years, many countries have introduced mandatory food fortification [55] and, according to the analysis of the published study results, it constitutes an effective measure of providing women with folic acid in the preconception period, as well as reducing the incidence of neural tube defects in children [10,56]. Available research shows that in European and Asian countries, for example, where there is no government legislation regulating food fortification with folic acid, spina bifida is much more common than in regions where this obligation exists [56].

According to studies by Kancherla (2018), Poland is one of the 71 countries in which the introduction of mandatory wheat flour fortification would help increase global measures aimed at the prevention of neural tube defects [57].

European countries are introducing obligatory fortification reluctantly. Health concerns arise from both folate deficiency and excess. Some studies point out that high consumption of folic acid may cause adverse health effects [58]. According to other authors dealing with neural tube defect prevention, the future will require balanced decisions in the scope of food fortification in all European countries [59].

In 2019, some members of the Polish Parliament asked “if Poland plans to introduce a national programme in order to reduce congenital defects through fortification of wheat flour with folic acid”. A government representative answered that there would not be any mandatory flour fortification and the government planned only educational campaigns on that matter [60].

A successful targeted primary prophylaxis depends on changes in the health behaviour of the female population of childbearing age. Such changes occur slowly and require systematic health education, as well as conducting interviews and surveys to estimate the extent to which women follow the recommendations.

In Poland, the implementation of the policy, which involves only voluntary fortification and prioritises educational measures taken by the sanitary and epidemiological stations within the Primary Prophylaxis of Neural Tube Defects programme, turned out to be an unsuccessful strategy.

In accordance with the studies presented above, women of childbearing age from southeastern Poland still do not have enough knowledge and practice on this matter, and it is not producing the desired results. Our analyses demonstrate that both proper education and providing women with basic information about the role of folic acid in the prevention of NTDs significantly increase the odds of this vitamin being taken before conception.

The development of modern medicine and studies on the influence of particular nutrients on the human body provides a great opportunity to ensure the healthy development of children, and thus prevent the consequences of measures not taken by unknowledgeable parents. However, to achieve this, proper education of the young generation is needed. In January, World Folic Acid Awareness Week is held. Unfortunately, it is not very popular in Poland.

### Limitations

The limitation of our study was in conducting the survey only among women studying at universities, and as such, the results can only be generalised to this group and the region of Poland subject to the study. However, the review of the results deriving from studies performed in other parts of Poland is consistent with our findings and may be indicative of the inefficacy of the recommendations for folic acid supplementation during the preconception period among women of childbearing age from other regions of Poland. The questionnaire did not collect socioeconomic status (SES) data. Further studies accounting for these limitations are necessary.

## 5. Conclusions

There is an urgent need to verify the foregoing nationwide measures, which involve recommendations for dietary supplementation with folic acid among Polish women of reproductive age, since, following the results of the studies, such measures are insufficient and not overly effective. Our findings indicate the need for the implementation of new measures consisting of active programmes and social campaigns in order to inform women about the daily intake of folic acid and its role in the prevention of NTDs, as well as to reduce the knowledge and health information deficit in the society. This requires strong educational measures, particularly in schools (for example during of physical education or biology classes). The results of this research can be used in planning regional preventive and education programs, e.g., through an appropriate information strategy. The education of female students should be particularly intensified in cooperation with university authorities, academic teachers, student governments, and general practitioners. As part of the regional health policy of the Podkarpackie Voivodeship, women studying at Podkarpackie institutions of higher education, as well as wider social groups, should be subject to monitoring, and activities undertaken should be systematically evaluated and periodically reported on the state of knowledge, frequency of consumption of folate-rich products, and folic acid supplementation.

## Figures and Tables

**Table 1:** Characteristics of the whole group (*N* = 1285).

		*N* = 1285	%
Age (year)	19–20	621	48.3
	21–25	491	38.2
	26–30	76	5.9
	31–35	51	4.0
	36–40	46	3.6
	22.27 ± 4.6		
Place of residence	village	702	54.6
	city	583	45.4
Number of children	0	1155	89.9
	1	78	6.1
	2	42	3.3
	3 and more	10	0.8
BMI classification *	Underweight	171	13.3
	Normal weight	965	75.1
	Pre-obesity	116	9.0
	Obesity class I	28	2,2
	Obesity class II	4	0.3
	Obesity class III	1	0.1
BMI (kg/m^2^)	21.55 ± 3.2		

* Body Mass Index (BMI) according to World Health Organization (WHO) criteria [33].

**Table 2:** Characteristics of the in the subgroup (*N* = 1127).

		*N* (1127)	%
Age (years)	21.0 ± 2.1		
Place of residence	village	639	56.7
	city	488	43.3
BMI classification *	Underweight	160	14.2
	Normal weight	854	75.8
	Pre-obesity	90	8.0
	Obesity class I	20	1.8
	Obesity class II	3	0.3
BMI (kg/m^2^)	21.4 ± 3.0		

* BMI according to WHO criteria [33].

**Table 3:** Folic acid supplementation among women without children and up to 30 years old.

Question	Answers	*N*	%
Do you supplement folic acid? (*N* = 1127)	yes	75	6.7
	no	1052	93.3
How often do you take folic acid? (*N* = 75)	every day	33	44.0
	a few times a week	14	18.7
	a few times a month	28	37.3
Why do you take folic acid? (*N* = 75)	own initiative	46	61.3
	Physician’s recommendations	27	36.0
	in case of becoming pregnant	1	1.3
	I want to become pregnant	1	1.3
Why do you not take folic acid? (*N* = 1052)	there is no need	515	49.0
	I know nothing about folic acid	226	21.5
	not important for me	58	5.5
	don’t know	253	24.0
Do you intend to take folic acid before getting pregnant? (*N* = 1052)	yes	547	52.0
	do not know	455	43.3
	no	50	4.8

**Table 4:** Comparison of the selected aspects of the knowledge among women using and not using supplements.

Knowledge of	Folic Acid Supplementation				*p*
	No (*N* = 1052)		Yes (*N* = 75)		
	*N*	%	*N*	%	
recommended dose (0.4 mg)	72	6.8	9	12.0	0.095
water-soluble vitamin	128	12.2	19	25.3	0.001
Food sources *					
green leafy vegetables	390	37.1	40	53.3	0.005
don’t know	403	38.3	16	21.3	0.003
role in the human body *					
prevention of neural tube defects	369	35.1	46	61.3	≤0.001
don’t know	510	48.5	5	6.7	≤0.001
source of knowledge *					
internet	550	52.3	42	56.0	0.533
school/university	244	23.2	22	29.3	0.226
TV	149	14.2	12	16.0	0.661
physician	243	23.1	29	38.7	0.002
close family	135	12.8	11	14.7	0.648
magazines/books	143	13.6	17	22.7	0.030
friends	89	8.5	12	16.0	0.027

*p*-value, probability calculated with the use of the chi-square test for independence. * The total percentage does not always add up to 100% as any number of response options could have been indicated.

**Table 5:** Logistic regression model showing the effect of folic acid knowledge factors on folic acid intake before pregnancy.

Independent Features	Do You Intend to Take Folic Acid before Getting Pregnant?	
	OR (95% CI)	*p*
knowledge of products rich in folates	1.84 (1.39–2.43)	≤0.001
prevention of neural tube defects	4.58 (3.39–6.18)	≤0.001
physician as a source of knowledge	1.68 (1.22–2.30)	0.002

−2log likelihood = 1274.5, df = 1248, ROC AUC = 0.73. *p*-assessment of the statistical significance of a given factor. OR–odds ratio (with a 95% confidence interval).

**Table 6:** Knowledge of neural tube defect (NTD) prevention and elements of specific knowledge about folic acid.

Selected Elements of the Specific Knowledge	Prevention of Foetal Neural Tube Defects				*p*
	No		Yes		
	*N*	%	*N*	%	
Liver	181	25.4	188	45.3	≤0.001
green leafy vegetables	188	26.4	242	58.3	≤0.001
citrus fruit	119	16.7	93	22.4	0.018
knowledge of recommended daily dose	18	2.5	63	15.2	≤0.001
physician as a source of knowledge	160	22.5	112	27.0	0.088

*p*—assessment of the statistical significance of a given factor.

## Data Availability

Data is contained within the article and Supplementary Materials.

## Supplementary Materials

The following are available online at https://www.mdpi.com/2072-6643/13/3/1001/s1, Supplement file 1: List of the survey questions; Table S1. The responses in the whole group (*N* = 1285); Table S2. The responses in the subgroup (*N* = 1127); Table S3. Self-assessment of the frequency of consumption of leafy vegetables in the group of examined women. Table S4. The reasons for not using folic acid supplementation vs. declared frequency of consumption of leafy green vegetables.
