# Supplementary material for: Effectiveness of Folic Acid Supplementation Recommendations among Polish Female Students from the Podkarpackie Region
Source: Nutrients. 2021 Mar 19;13(3):1001. doi: 10.3390/nu13031001 (PMC8003778; doi:10.3390/nu13031001)
Supplement: Supplementary file 1 [file nutrients-13-01001-s001.pdf]

File **1:** List of the survey questions

Section 1.

Q1. Have you ever heard the term "folic acid"?

- Yes
- No

Q2. What is "folic acid"? (only one option)

- water soluble vitamin
- fat soluble vitamin
- unsaturated fatty acid
- amino acid
- I don’t know

Q3. Please choose from the list those food products which are a rich source of folate (**folic acid**),(any number of responses)

- citrus fruits
- dark green leafy vegetables
- white bread
- apples
- liver
- cheese
- I don’t know

Q4. Do you take folic acid in supplement form? (only one option)

- Yes (**go to section 2)**
- yes, but only in the period when I am pregnant (**go to section 2)**
- no **(go to section 3)**

<u>Section 2</u>

Q1.2 How often do you take folic acid?

- every day
- a few times a week
- a few times a month

Q2.2. Why do you take folic acid?

- doctor’s recommendation
- self–initiative
- other

<u>Section 3</u>

Q1.3. If you do not take folic acid, why?

- I do not feel such a need
- it is not important to me
- I don’t know anything about folic acid
- I don’t know

Q2.3. Do you intend to take folic acid when planning pregnancy?

- Yes

- No
- I don't know

Q5. What is the recommended daily dose of folic acid for women of reproductive age (in Poland)?

- 0.4 mg
- 0.5 mg
- 0.8 mg
- 1 mg
- I don't know

Q6. What role do you think folic acid plays in the human body? (any number of responses)

- lowers homocysteine levels
- prevents neural tube defects in the fetus
- contributes to regulation of development and maturation of red blood cells
- is an important factor in the prevention of cardiovascular diseases
- I don't know

Q7. What is your main source of knowledge about folic acid: (any number of responses)

- doctor (physician)
- school / college
- Internet
- TV
- magazines / books
- immediate family
- friends
- other
- does not apply

Q8. How often do you eat the dark-leafy vegetables (e.g. broccoli, spinach, etc.)

- every day
- 2-3 times a week
- once a week
- never

Table S1. The responses in the whole group (N=1285)

Question	Answers	N=1285	%
Q1.Have you ever heard the term 'folic acid'?	yes	1157	90.0
	no	128	10.0
Q2.What is folic acid?	fat soluble vitamin	140	10.9
	water soluble vitamin	179	13.9
	amino acid	158	12.3
	unsaturated fatty acid	144	11.2
	I don't know	664	51.7
Q3.Please choose from the list those food products which are a rich source of folate (folic acid) *	dark green leafy vegetables	534	41.6
	liver	417	32.5
	citrus fruits	242	18.8
	apples	101	7.9
	cheese	76	5.9
	white bread	33	2.6
	I don't know	450	35.0
Q4.Do you take folic acid in supplement form?	yes	90	7.0
	Yes, but only when I am pregnant	89	6.9
	no	1106	86.1
Q5.What is the recommended daily dose of folic acid for women of reproductive age (in Poland)?	0.4 mg	114	8.9
	0.5 mg	103	8.0
	0.8 mg	80	6.2
	1 mg	42	3.3
	I don't know	946	73.6
Q6.What role do you think folic acid plays in the human body?*	prevention of neural tube defects in the fetus	522	40.6
	regulation of development and maturation of red blood cells	311	24.2
	prevention of cardiovascular diseases	118	9.2
	lowers homocysteine levels	53	4.1
	I don't know	542	42.2
Q7.What is your main source of knowledge about folic acid?*	Internet	681	53.0
	physician	349	27.2
	school / college	290	22.6
	magazines / books	204	15.9
	TV	175	13.6
	immediate family	157	12.2
	friends	122	9.5
	other	74	5.8
	does not apply	226	17.6
Q8. How often do you eat the dark-leafy vegetables (e.g. broccoli, spinach, etc.)?	every day	48	3.8
	2-3 times a week	318	24.7
	once a week	713	55.5
	never	206	16.0

* any number of responses

Table S2. The responses in the subgroup (N=1127)

Question	Answers	N=1127	%
Q1.Have you ever heard the term 'folic acid'?	yes	1004	89.1
	no	123	10.9
Q2.What is folic acid?	fat soluble vitamin	122	10.8
	water soluble vitamin	147	13.0
	amino acid	142	12.6
	unsaturated fatty acid	124	11.0
	I don't know	592	52.5
Q3.Please choose from the list those food products which are a rich source of folate (folic acid) *	dark green leafy vegetables	430	38.2
	liver	369	32.7
	citrus fruits	212	18.8
	apples	82	7.3
	cheese	68	6.0
	white bread	29	2.6
	I don't know	419	37.2
Q4.Do you take folic acid in supplement form?	yes	75	6.7
	Yes, but only when I am pregnant	-	-
	no	1052	93.3
Q5.What is the recommended daily dose of folic acid for women of reproductive age (in Poland)?	0.4 mg	81	7.2
	0.5 mg	94	8.3
	0.8 mg	70	6.2
	1 mg	36	3.2
	I don't know	846	75.1
Q6.What role do you think folic acid plays in the human body?*	prevention of neural tube defects in the fetus	415	36.8
	regulation of development and maturation of red blood cells	245	21.7
	prevention of cardiovascular diseases	105	9.3
	lowers homocysteine levels	45	4.0
	I don't know	515	45.7
Q7.What is your main source of knowledge about folic acid?*	Internet	592	52.5
	physician	272	24.1
	school / college	266	23.6
	magazines / books	161	14.3
	TV	160	14.2
	immediate family	146	13.0
	friends	101	9.0
	other	69	6.1
	does not apply	215	19.1
Q8. How often do you eat the dark-leafy vegetables (e.g. broccoli, spinach, etc.**)?**	every day	42	3.7
	2-3 times a week	272	24.1
	once a week	634	56.3
	never	179	15.9

* any number of responses

Table S3. Self-assessment of the frequency of consumption of leafy vegetables in the group of examined women.

Frequency of consumption of leafy greens vegetables	Supplementation with folic acid (p = 0.334)					
	Yes		No		Total	
	N	%	N	%	N	%
Every day	42	4.0	0	0.0	42	3.7
2-3 times a week	254	24.1	18	24.0	272	24.1
Once a week	591	56.2	43	57.3	634	56.3
Never	165	15.7	14	18.7	179	15.9

Table S4. The reasons for not using folic acid supplementation vs. declared frequency of consumption of leafy green vegetables.

Frequency of consumption of leafy greens vegetables	Why do you not take folic acid? (p = 0.896)							
	there is no need		I know nothing about folic acid		not important to me		I don't know	
	N	%	N	%	N	%	N	%
Every day	23	4.5	8	3.5	4	6.9	7	2.8
2-3 times a week	124	24.1	51	22.6	13	22.4	66	26.1
Once a week	292	56.7	127	56.2	32	55.2	140	55.3
Never	76	14.8	40	17.7	9	15.5	40	15.8
